# Emerging Nuclear Medicine Imaging of Atherosclerotic Plaque Formation

**DOI:** 10.3390/jimaging8100261

**Published:** 2022-09-27

**Authors:** Anton Kondakov, Alexander Berdalin, Mikhail Beregov, Vladimir Lelyuk

**Affiliations:** 1 Ultrasound and Functional Diagnostics Department, Federal Center of Brain Research and Neurotechnologies, 117513 Moscow, Russia; 2 Radiology and Radiotherapy Department, Pirogov Russian National Research Medical University, 117997 Moscow, Russia

**Keywords:** atherosclerosis, plaque, molecular imaging, preclinical, radiotracers, cardiovascular, pathophysiological

## Abstract

Atherosclerosis is a chronic widespread cardiovascular disease and a major predisposing factor for cardiovascular events, among which there are myocardial infarction and ischemic stroke. Atherosclerotic plaque formation is a process that involves different mechanisms, of which inflammation is the most common. Plenty of radiopharmaceuticals were developed to elucidate the process of plaque formation at different stages, some of which were highly specific for atherosclerotic plaque. This review summarizes the current nuclear medicine imaging landscape of preclinical and small-scale clinical studies of these specific RPs, which are not as widespread as labeled FDG, sodium fluoride, and choline. These include oxidation-specific epitope imaging, macrophage, and other cell receptors visualization, neoangiogenesis, and macrophage death imaging. It is shown that specific radiopharmaceuticals have strength in pathophysiologically sound imaging of the atherosclerotic plaques at different stages, but this also may induce problems with the signal registration for low-volume plaques in the vascular wall.

## 1. Introduction

Atherosclerosis is a frequent disorder in today’s population that leads to ischemic syndromes in many vascular areas, including ischemic stroke and myocardial infarction, which are the leading causes of death in Russia and around the world [1,2]. At the same time, atherosclerosis affects people of different age groups who have never had a cardiovascular event in their lives. Consequently, modern researchers are challenged with not only detecting atherosclerosis but also predicting the risk of future fatal and non-fatal vascular events. To solve such issues, non-invasive diagnostic techniques might be employed successfully.

Nuclear medicine imaging, a method of molecular imaging, which can produce anatomical and functional images of different metabolic processes in the human body, is a promising method to investigate processes of plaque formation, vulnerability, and rupturing. Radiopharmaceuticals (RPs) have virtually no side effects, with the only serious contraindication to their use being pregnancy [3]. Nuclear medicine imaging is based on the external registration of the radiation emitted by the RP injected and distributed in the human body. It allows us to trace different pathophysiologic processes in the object (i.e., a laboratory animal). Three major techniques are used in nuclear medicine currently: (1) Scintigraphy, (2) single photon emission computed tomography (SPECT), and (3) positron emission tomography (PET). The first two are based on the detection of one photon for each decay event with the special type of planar detector (gamma-camera) that may rotate around the object and produce multiple projections in order to generate a set of tomographic images. The basis of PET is a concurrent registration of two photons that appear after the annihilation of a positron emitted by radiopharmaceutical. PET has some advantages over SPECT, such as higher spatial resolution and the ability of quantitative assessment of the image.

Similar reviews were published earlier [4,5,6], but since then, new data appeared, and new studies have been published, which makes it valuable to update information.

In our previous review, we discussed radiopharmaceuticals that are available for the clinical practice of plaques and the role of vulnerable plaque in major cardiovascular events [7]. The goal of this review is to analyze published studies of radiopharmaceuticals targeted at components of the atherosclerotic process but still described only in experimental or small-scale clinical studies.

## 2. Evolution of an Atherosclerotic Plaque

To describe the ways of using nuclear medicine techniques in the imaging of pathological processes in atherosclerosis, it is necessary to consider the main pathophysiological mechanisms mediating the formation of plaques and their destruction.

Atherosclerosis is a condition mediated by immune mechanisms, which is realized by the accumulation of lipoproteins in the wall of arterial vessels, leading to its focal thickening and the formation of atherosclerotic plaques in medium- and large-caliber arteries. Lipids, inflammatory infiltrates, smooth muscle cells, and connective tissue composes an atherosclerotic plaque, and a fibrous cap covers it. Damage to the latter leads to the fact that the internal contents of the plaque interact directly with the blood, which can cause thrombosis, and in the case of fragmentation of both the plaque and the blood clot on its surface—embolism in the distal vascular bed [8].

### 2.1. Fatty Streaks

Plaque evolution begins with the stage of “fatty streaks”, which are formed already in childhood and adolescence due to the accumulation of low- and very-low-density lipoproteins, their binding to proteoglycans in the intima, and the development of an inflammatory reaction leading to endotheliocyte activation. In the early stages, an increase in endotheliocyte permeability to low-density lipoproteins rich in cholesterol is observed, as well as an increase in the adhesive properties of the endothelial surface, which promotes monocyte migration from the vascular lumen to the subendothelial space [9]. The driver of increasing the adhesive properties of the endothelium is the adhesion molecules expressed on the cell surface, among which VCAM-1 and ICAM-1 (from the immunoglobulin superfamily) and P-selectins play a special role [10].

#### 2.1.1. Labeled Lipoproteins

Visualization of fatty streaks in vivo is not important from the point of view of clinical practice: Timely lifestyle modification and normalization of lipid metabolism, according to Pitman et al. [11], may lead to their regression and reduce the risk of developing atherosclerosis in the future [12]. Nevertheless, radioactive labels containing low-density lipoproteins have been actively used to study lipid metabolism.

In particular, in 1988, the work of Vallabhajosula et al. was published, which compared radioactive labels for low-density lipoproteins and concluded that technetium-99m (^99m^Tc) is preferred as such a label [13]. Vallabhajosula et al. have also demonstrated that in patients with hypercholesterolemia, ^99m^Tc-labeled LDL is captured by actively developing atherosclerotic plaques and xanthomas that contain foam cells and macrophages [14]. Indium-111 (^111^In) and gallium-68 (^68^Ga) isotopes were also used as labels for LDL [15]. Rosen et al. found that the absorption of ^111^In-LDL in the proximal atherosclerotic aorta of rabbits with hypercholesterolemia was 2.5 times higher than in healthy animals [16]. Pirich and Sinzinger studied LDL metabolism in humans using radioactive iodine-123 and scintigraphy during therapy with isradipine and alprostadil [17]. In their study, in particular, it was demonstrated that the uptake of isotope-labeled LDL in atherosclerotic lesions provides valuable information for monitoring the natural course of the process, as well as evaluating the effectiveness of various types of interventions. Thus, the authors explained the anti-atherosclerotic activity of isradipine by its ability to stimulate the production of vascular prostaglandin IL-2, which leads to an increase in cAMP content and an increase in the action of cholesterol esterase. Moreover, Pietzsch et al. reported the development of fluoro-18 (^18^F)-labeled native and oxidized forms of LDL [18], which can be used in PET studies of fatty acid metabolism.

A few years ago, Pérez-Medina et al., using high-density lipoproteins labeled with zirconium-89 (^89^Zr), demonstrated that HDL accumulation in plaques is also increased [19]. In this study, it is stated that this isotope of zirconium is the preferred label for tracking lipid metabolism by PET due to its long half-life (78 h), which allows its accumulation in biological objects to be recorded for a long time. Studies were conducted on models of atherosclerosis in animals, including mice, rabbits, and pigs, and showed increased uptake of the studied radiopharmaceutical in the aorta compared with control animals without atherosclerosis. At the same time, the observed migration of labeled HDL particles into lipid plaques with signs of inflammation means that such particles are accumulated high-risk atheromas. Moreover, labeled HDL particles are recognized by the authors as promising radiopharmaceutical not only for visualization but also as a drug delivery system that transports drugs located in its core directly to those plaques that require therapy. In the editorial on the study of Pérez-Medina et al., it was indicated that although the particles, labeled with ^89^Zr, are unlikely to be available for studies in humans due to the long half-life of ^89^Zr (78 h) and, thus, large absorbed doses of radiation, the development of this technique may accelerate the stream of technology with the use of labeled HDL particles through the preclinical stage and to obtain important information about the effects of drugs on HDL metabolism [20].

#### 2.1.2. Oxidized LDL

Oxidized LDL that is accumulated in the vessel wall is an attractive target for molecular imaging. Due to the fact that ex vivo monoclonal antibodies specific to oxidized forms are used for ex vivo staining of LDL, Tsimikas suggested that they also can be used in vivo to visualize the progression or regression of atherosclerotic lesions [21]. Plaque uptake of iodine-125 (^125^I)-labeled monoclonal antibodies has been shown to strongly correlate with the degree of atherosclerosis, measured as a percentage of the surface area or weight of the aorta, and thus provides an accurate quantitative assessment of the atherosclerotic load. In vivo scintigraphy with mouse antibodies to oxidized lipid forms has shown that visualization of atherosclerotic lesions is possible using this technique [21].

A potential target for molecular imaging of atherosclerosis may be oxidation-specific epitopes (OSE) since they exist at the intersection of oxidative stress, lipid metabolism, and inflammation. Lipid oxidation leads to the formation of highly reactive products that generate structural neoepitopes that are recognized by the body’s own immune system. Epitopes present on oxidized LDL stimulate their uptake by macrophages, contributing to their transformation into foam cells and the development of inflammation in the focus of atherosclerotic lesions. In addition, elevated concentrations of circulating epitopes of this type are associated with an increased risk of cardiovascular diseases [22]. Accordingly, OSE detection can be useful in objectifying the inflammatory process, which is especially important in atherosclerosis [23].

In 2002, the first human antibody capable of recognizing a unique epitope specific for oxidation was developed, which can also be labeled with appropriate labels for use in nuclear, magnetic resonance, or ultrasound imaging [21], though the development of such antibodies was initiated in 1990 [24]. The use of specific antibodies was demonstrated in laboratory animals, using the isotopes iodine-131 (^131^I) [25] and ^125^I [26] as labels. These methods were not used in the clinical setting.

To develop specific drugs, a library of antigen-binding fragments (Fabs) from human fetal cord blood was created in 2018. After several rounds of screening against the malondialdehyde-acetaldehyde (MAA) epitope, a specific fragment named LA25 was identified as an attractive candidate for creating RPs due to its specificity for the named epitope [27]. The study showed that LA25 specifically binds to MAA-carrying LDL and significantly inhibits their binding to macrophages. In tests performed on human coronary samples, LA25 was present in minimal amounts in areas with abnormal intimal thickening but accumulated in significant amounts in late forms of fibroatheroma and damaged plaques. PET labeling isotope, ^89^Zr, was used to label these antibodies. After an injection to ApoE-deficient mice, radioactivity in aortic plaques was more than three times higher for ^89^Zr-LA25 compared to the control Fab, ^89^Zr-LA24, as assessed by ex vivo autoradiography of the resected mouse aorta. In addition, the activity of ^89^Zr-LA25 was localized in areas rich in macrophages [26]. In the next stage, the authors used the indicator to visualize atherosclerotic lesions in larger animals. Using PET/MR, they observed a 32% increase in the uptake of the radioactive tracer in the aorta of rabbits with atherosclerosis compared to control animals. In addition, the accumulation of the radioactive indicator correlated with the area of the affected vessel wall, macrophage staining, and lipid content. It should be noted that the indicator virtually did not accumulate in the myocardium [27]. These in vivo observations support the assumption that OSE-rich plaques in humans can be visualized not only in the aorta but also in the coronary arteries due to the low accumulation of RP in the myocardium [23]. In a recent work by Zhang et al., oxidation-specific epitope imaging was described by means of other methods such as near-infrared and magnetic resonance imaging, which provides the possibility of plaque imaging with no radiation burden [28].

Directly oxidized LDL was also used as a radioactive label. In particular, Iuliano et al. found that ^99m^Tc-labeled LDL is eliminated from the blood faster than native LDL, and its absorption in the liver is more intense. In addition, scintigrams of patients with atherosclerotic carotid artery disease showed significantly greater uptake of oxidized LDL forms in plaque compared to adequate controls [29]. Studies of oxidized LDL metabolism continue to date, including the use of ^123^I as a radioactive label [30], but the clinical significance of this radiopharmaceutical has not yet been determined.

#### 2.1.3. Adhesion Molecules

Immunoglobulins and P-selectin expressed on the endothelial surface are promising potential targets at the considered stage of pathogenesis. For VCAM-1 visualization, Nahrendorf et al. developed an ^18^F-labeled compound ^18^F-4V, which is a linear tetrapeptide with a high tropicity to the expressed protein [31]. Later, Broisat et al. developed ^99m^Tc-labeled nanobodies, cAbVCAM1-5. The latter are antibodies that contain only heavy chains, which are naturally present only in camels and are the smallest possible (10–15 kDa) functional immunoglobulin-like antigen-binding fragment [32,33]. In 2016, Bala et al. published a paper on the production of such nanobodies labeled with fluorine-18, which makes them suitable for use in PET/CT [34]. Regardless of the radioactive isotope used, the researchers managed to achieve high specificity of the developed tracers: The accumulation of RP in murine aortic plaques with signs of inflammation significantly exceeds that in normal tissues. However, VCAM-1 molecules are specific not only for atherosclerosis, but they are also expressed in any other inflammatory processes accompanied by endothelial activation, which leads to false-positive results. In addition, studies of these drugs were carried out mainly on laboratory animals and were not evaluated in clinical trials until the present moment, so their use outside the framework of clinical trials in humans is currently impossible.

VCAM-1 is present on the surface of cell membranes of the endothelial lining covering fibrous-cap atheromas and lipid-rich atheromas, as well as endotheliocytes of the new vasa vasorum, smooth muscle cells, and macrophages, according to Davies et al. [35]. According to O’Brien et al. [36], VCAM-1 expression on the smooth muscle cell membrane is significantly increased in atherosclerotic lesions of human coronary vessels, whereas its presence on the vascular endothelium is low in both the affected and control parts, which is associated with newly formed vessels of the vascular wall. Thus, the potential application of radioligands to VCAM-1 exists not only at the stage of lipid spots but also in later stages of atherosclerotic lesions. This explains the growing interest in the VCAM-1 imaging—in 2021, two new radiopharmaceuticals for this purpose were described by Pastorino et al. [37].

Two preclinical studies have focused on visualization based on labeled molecules that are tropic to P-selectin. In the work of Nakamura et al. radionuclide copper-64 (^64^Cu) was used, which was introduced as a label for monoclonal antibodies to P-selectin [38]. The distribution of such antibodies was studied using PET/CT, and histological examination of the aorta with autoradiography showed that antibodies selectively accumulated in atherosclerotic plaques. ^64^Cu has an important advantage of a long half-life, which is 12.7 h, which allows for studying slow biological processes and delayed imaging [39]. On the other hand, a longer half-life will result in a larger absorbed dose in the human body.

In another paper on visualization based on P-selectin binding, published by Li et al. [40], ^68^Ga-labeled fucoidan, a natural P-selectin ligand expressed on leukocytes and found in vivo as one of the products of brown algae processing, was chosen as the RP [41]. In his work, Li et al. convincingly showed that a significant accumulation of the radiopharmaceutical developed by them occurs in atherosclerotic lesions, in which high macrophage density and P-selectin expression are observed, while “inactive” atherosclerotic lesions accumulated RP to a much lesser extent. It is also important that the results of in vivo imaging were compared with tomograms obtained on an MRI with a magnetic field induction of 17.6 T, as well as the results of autoradiographic and histological analysis [40].

Recently, new probes targeted against P-selectin and VCAM-1 have been being at the stage of development. Like in some other targets, there is a shift to non-ionizing imaging of the inflamed endothelium and risk stratification of atherosclerosis with the use of dual-targeted microparticles of iron oxide and MRI [42].

The small size of fatty streaks causes difficulties in their visualization, which are associated with insufficient accumulated radioactivity in the vessel wall, low resolution of diagnostic equipment, and high radioactivity of the blood pool. Assessment of the accumulation of most of the noted RPs was carried out at the stage of formed atherosclerotic plaque, reflecting the activity of inflammatory reactions in its structure and, thus, the possible vulnerability of the atheroma.

It should be noted that all the above-mentioned RPs can be used to assess atherosclerotic changes at later stages of their development since the processes of lipid accumulation and inflammatory activation of the endothelium in plaque evolution continue constantly.

### 2.2. Macrophage Migration

The next step in the formation of atherosclerotic plaque is the accumulation of macrophages in the arterial walls. Upon entering the structure of the fatty streak, blood monocytes transform into macrophages and absorb lipoproteins, turning into xanthomous (or foam) cells. Extracellular proteoglycans secreted by smooth muscle cells also progressively bind lipids. The necrosis of macrophages and smooth muscle cells leads to the formation of necrotic residues and supports inflammation. The accumulation of extracellular lipids pooling together and resulting in cell necrosis also increases. Gradually, this leads to a distortion of the normal architecture of intima. Increasing pools form lipid-rich necrotic nuclei, which are usually located in the central part of the intima, and eventually occupy from 30% to 50% of the arterial wall volume [8,9].

At this stage, the main objects for visualization are macrophages expressing somatostatin type 2 receptors (SSTR2), mannose receptors (MR), folic acid receptors (FR), C-X-C chemokine receptors of type 4 (CXCR4), as well as their proliferation and increased glycolytic activity, and proteases secreted by them. In addition, molecular imaging of other white blood cells expressing an antigen associated with white blood cell function (LFA-1), specific markers of inflammation (translocation protein 18 kDa, TSPO, formerly known as the peripheral benzodiazepine receptor), markers of oxidized LDL accumulation (their LOX-1 receptor) are possible.

#### 2.2.1. Migration of Native Monocytes

Binding to adhesion molecules on the surface of activated endotheliocytes, monocytes migrate to the area of the atherosclerotic lesion and accumulate there, making it possible to visualize using RPs. Radiolabeling a person’s peripheral blood cells, including monocytes, is a well-known procedure. Most often, blood leukocytes are labeled in this way to assess inflammatory foci using oxine combined with ^111^In or ^99m^Tc-exametazime [43].

Kircher et al. have demonstrated the ability to confirm the recruitment of ^111^In-oxy-quinoline (^111^In-oxine)-labeled monocytes into existing atherosclerotic lesions in apolipoprotein E-deficient mice using single-photon emission computed tomography. The long half-life of ^111^In (2.8 days) allows detection of monocytes within a week after administration, and anatomical data obtained by combined CT allows localization of hot spots of monocyte infiltration in the submillimeter range [44]. It is important to note that injecting a radioactive tracer had no effect on monocyte viability and that cell accumulation primarily occurred in the ascending aorta, which was the site of the highest plaque density in most experimental animals. Some small foci did not emit a sufficient signal for detection, which could be both the result of different lesion activity (some plaques may be more “active” than others) and the fact that small plaques did not accumulate enough cells to exceed the detection threshold for PET. The authors concluded that the method can be successfully used to detect lesions that recruit significant numbers of monocytes and are more “active” [44]. ^111^In-oxine is approved for use both in Europe and in Russia and is a promising tracer for cell labeling [45].

Data concerning the use of ^111^In-labeled monocytes are limited to small studies [46,47]. There are no clinical studies to recommend this method for use in general clinical practice.

#### 2.2.2. Glucose Metabolism

Fluorodeoxyglucose (^18^F-FDG) is an ^18^F-labeled glucose analog, therefore, its uptake by tissues is a marker for the tissue uptake of glucose.

As a consequence of chemical similarity with glucose, ^18^F-FDG accumulates in macrophages in atherosclerotic plaque, which utilize glucose as an energy source and have high GLUT-1 and -3 expression. This phenomenon is most pronounced in hypoxic areas where it compensates for inefficient glucose utilization [48]. The possibilities and limitations of this technique were discussed in a previous study [7].

#### 2.2.3. Cell Membranes

Choline incorporation into the cell membrane is a multi-stage process, the main stages of which include entering the cell via specific transport mechanisms, phosphorylation by choline kinase, and conversion to phosphatidylcholine [49]. This technique was discussed in a previous study [7].

#### 2.2.4. Scavenger Receptors

Macrophages, getting into the artery wall with oxidized LDL contained in its structure, trigger a pro-inflammatory reaction. Macrophages recognize modified LDL by using toll-like and scavenger receptors. For example, CD36, a scavenger receptor, is able to recognize oxidized LDL and associate with a toll-like receptor that triggers pro-inflammatory signaling [50]. Non-specific visualization of such scavenger receptors can be achieved using acetylated forms of LDL, which was demonstrated by Gurudutta et al. [51]. Specific ligands—antibodies—were synthesized for MR imaging of macrophage scavenger receptors (MSRs) [52], which creates prospects for the future synthesis of radiolabels based on them.

Another scavenger receptor, LOX-1 (a lectin-like receptor for oxidized low-density lipoproteins), is a promising method for determining macrophage activity in atherosclerotic artery walls. Ishino et al. developed the RP based on ^99m^Tc-labeled monoclonal antibodies specific to this receptor. The level of RP accumulation in active atheromas turned out to be higher than in neointimal or other, more stable processes, and therefore the authors of the study concluded that visualization of LOX-1 expression can be a useful tool for predicting a high risk of atheromas [53]. The results of a large-scale study comparing SPECT/CT and MR imaging of the distribution of antibodies to LOX-1 have also been published, which showed that radionuclide studies using these antibodies register foci of high accumulation mainly in the aortic arch of mice affected by atherosclerosis [54].

Other types of scavenger receptors may potentially be used for atherosclerotic plaque inflammation imaging, such as ^68^Ga labeled anti-CD163-antibody. It was reported by Eichendorff et al. [55] that the specific radiotracer—^68^Ga-ED2 specifically binds CD163 in vitro and in vivo and is applicable for inflammatory disease imaging. This scavenger receptor has high expression in a subpopulation of the alternatively activated M2 macrophages called hemorrhage-associated macrophages that are found in hemorrhagic zones of atherosclerotic plaques [56]. Thus, the use of ^68^Ga-ED2 in atherosclerosis may predict the source of plaque instability other than inflammation—the hemorrhage. It was also shown by Bigalke et al. that another scavenger receptor, CD68, expressed by activated macrophages, plays a crucial role in the development of atherosclerotic plaques as it binds low-density lipoproteins, which leads to foam cells formation [57]. This was observed by means of PET/CT with a specific radiopharmaceutical ^64^Cu-CD68-Fc.

#### 2.2.5. Somatostatin Receptors

Visualization of the RP targeted to somatostatin receptors was discussed earlier [7].

In addition to clinical application, in a recent preclinical study by Meester et al., it was revealed that ^111^In-DOTA-JR11 can be used for SPECT imaging of somatostatin SSTR2 receptors. Noticeably, DOTA-JR11 may have an advantage over DOTA-TATE since its higher uptake [58]. Moreover, a new RP was recently described for neuroendocrine tumor imaging—^68^Ga-DOTA-JR11, which makes it possible to use the same ligand for PET imaging [59], although we were unable to find any mention of its application for plaque or inflammation imaging.

#### 2.2.6. Mannose Receptor

Another possible target for detecting activated macrophages in vulnerable plaque is the mannose receptor (a type C lectin located on the surface of macrophages, dendritic cells, and some other cells that recognize terminal mannose residues). Imaging based on binding to this type of receptor was developed to identify sentinel lymph nodes in tumors [60], but it has also found application in atherosclerosis studies. In particular, in the experimental work, the results of which were published by Varasteh et al., accumulation of this ^111^In-labeled ligand occurred in the vessels of mice affected by atherosclerosis and coincided in localization with macrophage accumulations according to histological and immunohistochemical studies [61]. Varasteh et al. have published the results of a new study on ^68^Ga-labeled mannose-receptor-specific antibodies, which are also shown to be useful in assessing plaque stability and determining their vulnerability [62]. In a clinical trial, Zanni et al. used ^99m^Tc-labeled tilmanocept as a method for visualizing macrophages in the vascular wall, which was administered to HIV-infected patients with imaging of its accumulation in the aortic wall [63]. This RP is approved for use by the American Food Drug Administration (FDA) as an oncological imaging agent. Both in the described in vivo study and in experiments ex vivo, RP accumulation was demonstrated in areas with a high concentration of macrophages carrying the mannose receptor. The accumulation of RP in the aortic wall of HIV-infected individuals was higher (20.4% vs. 4.3% in the control group) and corresponded mainly to the areas of non-calcified atherosclerotic plaques according to CT angiography [63].

For specific molecular imaging of mannose receptors, RP ^18^F-fluoro-D-mannose (FDM) is also used. This glucose isomer is identically transported through glucose receptors but with a more specific accumulation profile in anti-inflammatory (M2-like) macrophage populations. When analyzing the results of the study in model animals, in comparison with ^18^F-FDG, ^18^F-FDM increased the level of detection of inflammation in plaques with higher RP uptake (by 35%) [64]. Another developed compound based on ^68^Ga-labeled human serum albumin enriched with mannose (abbreviated as ^68^Ga-NOTA-MSA) binds to the same receptor. Studies of its usage in vivo on laboratory animals have shown a high affinity to the population of anti-inflammatory (M2-type) macrophages, which are more characteristic of unstable plaques [65]. At the same time, Bobryshev et al. noted that macrophages with anti-inflammatory secretion, including M2-type, are more often found in stable and regressing plaques [50].

#### 2.2.7. Folate Receptor

Folate receptors are detected on the membranes of many human cells, including activated macrophages [66,67]. The applicability of their ligands in laboratory animals (mice) with atherosclerosis was studied by Ayala-Lopez et al. [68], in which ^99m^Tc-labeled etarfolatide (^99m^Tc-EC20), which is tropic to β-type folate receptors located on the macrophage surface, was used as a radioactive label. It was shown that in these model animals, increased fixation of RP occurs in the aortic arch, where the greatest number of atherosclerotic plaques occur. The authors of the study noted that activated macrophages carrying a folate receptor on the surface synthesize pro-inflammatory cytokines and can reasonably be considered a promising marker of the vulnerability of the plaques.

There is also information about the development of an RP for PET based on ^18^F-labeled fluorofolic acid [69], the effectiveness of which was confirmed in ex vivo experiments, but this tracer has not been evaluated in preclinical and clinical practice. A similar RP, which is ^18^F-labeled folate bound to the radiolabel via a binding molecule, was developed and tested in vivo by Silvola et al. [70]. Researchers confirmed its ability to accumulate in the plaques of the arteries of model animals, and the background capture of RP by the myocardium was minimal and did not interfere with the visualization of plaques in the coronary arteries, which radically distinguished it from ^18^F-FDG.

#### 2.2.8. TSPO

The 18 kDa translocation protein, or TSPO, formerly known as the peripheral benzodiazepine receptor, is found on the membranes of activated macrophages. An RP known as Carbon-11(^11^C)-PK11195 was developed for this receptor. In a clinical trial conducted by Pugliese et al., its increased accumulation in the arterial wall in vasculitis has been demonstrated [71]. When assessing intra-plaque inflammation in 32 patients in the study of Gaemperli et al., it was shown that in cases with a recent ischemic event, ipsilaterally located plaques had a lower X-ray density (according to CT data) and an increased accumulation of ^11^C-PK11195 [72].

The short half-life of ^11^C (20 min) makes it necessary to develop an RP with a longer half-life. These include the radioligand to TSPO, called ^18^F-GE-180. It was applied by Hellberg et al. in a series of preclinical experiments, and as a result, it was found that ^18^F-GE-180 demonstrates macrophage uptake not only in atherosclerotic plaques but also in unchanged arterial walls in mice. In general, the accumulation in atherosclerotic lesions did not exceed that in the intact arterial wall, and consequently, ^18^F-GE-180 did not demonstrate any advantages in verifying inflammation in plaques compared to previously studied radiolabels for TSPO [73].

The small-scale clinical trial conducted by Schollhammer et al. reported other tracers for TSPO imaging—^11^C-PBR28 and ^18^F-PBR06. The authors of the study conclude that these tracers are not viable clinical tools for imaging inflammatory vascular disease, although they show good uptake on surgical samples in vitro [74].

#### 2.2.9. Chemokine Receptors

Many different RPs can reasonably be used to detect increased expression of chemokine receptors. Non-specific to this type of receptor is RP ^64^Cu-DOTA-vMIP-II, which demonstrated high affinity to atherosclerotic plaques in mechanically induced atherosclerosis in vivo in a study conducted by Liu et al. [75].

Another RP for chemokine receptor imaging, ^68^Ga-pentixaphor, binds with nanomolar affinity to the CXCR4 receptor, which is found on the surface of inflammatory cells. In a study by Hyafil et al., accumulation of this RP was demonstrated using PET/MR in the aortic arch and right carotid artery, in which atherosclerosis in the studied rabbits was mechanically induced. The right-sided RP fixation significantly exceeded that in the left carotid artery [76].

Li et al. showed that the accumulation of RP detected by PET/MR with ^68^Ga-pentixafor in the arterial walls of patients correlates with an increased frequency of the main risk factors for cardiovascular events, which indicates the potential use of this RP in the clinical diagnosis of atherosclerosis [77]. Similar data was published by Weiberg et al. based on the results of a retrospective study [78]. In 92 patients with atherosclerosis, 652 plaques were identified by ^68^Ga-pentixafor imaging and compared head-to-head with ^18^F-FDG PET results in a study by Kircher et al. [79]. The results indicate that ^68^Ga-pentixafor shows more lesions with a higher uptake of the RP, but only a weak correlation between the two tracers was achieved. Both tracers correlate negatively with plaque calcification [79]. This may be explained by the fact that not only macrophages, but other cells (like T-cells and smooth muscle cells), involved in the atherosclerotic process are presenting chemokine receptors on their surface [80].

Clinical evaluation of the ^68^Ga-pentixafor for atherosclerotic lesions is thoroughly described in a newly published study by Lu et al., with a head-to-head comparison with ^18^F-FDG. As it was shown earlier, ^68^Ga-PentixaFor PET/MRI identified many more lesions than ^18^F-FDG PET/MRI, and patients with high-risk cardiovascular factors exhibit an increased uptake of this specific RP [81].

Another chemokine receptor, CCR2, may be visualized with a specific RP named ^64^Cu-DOTA-ECL1i, which was demonstrated by English et al. in their work on abdominal aorta aneurism [82]. It is likely that this RP can also be used to visualize atherosclerotic plaques.

#### 2.2.10. LFA-1

Recently, radiopharmaceuticals that target the lymphocyte-function associated antigen-1 (LFA-1) have appeared. This antigen is found on the surface of lymphocytes and some other white blood cells and binds to ICAM receptors, playing a role in the adhesion and transmission of a pro-inflammatory signal [83]. For its visualization, the radiopharmaceutical ^111^In-DOTA-butylamino-NorBIRT (DANBIRT), used in SPECT, was developed. Meester et al. showed that the uptake of this RP correlates with the presence of CD68-expressing macrophages and LFA-1-expressing inflammatory cells in the atherosclerotic plaque. In addition, in an ex vivo study, the uptake of DANBIRT by human carotid plaque correlates with the presence of macrophages expressing CD68 and inflammatory cells expressing LFA-1, which indicates the potential of DANBIRT for non-invasive visualization of atherosclerotic plaque inflammation [84]. Its uptake is negatively correlated with the volume of plaque calcification, which allows us to conclude that a plaque is stable when it demonstrates significant uptake [85]. The use of the drug has been described in preclinical practice with a positive result in detecting atherosclerosis [86], but the radiopharmaceutical has not been studied in clinical practice yet.

#### 2.2.11. Nicotinic Acetylcholine Receptor

^18^F-ASEM, that is α7-nicotinic acetylcholine receptor targeted RP, could play a complementary diagnostic role in vulnerable atherosclerotic plaque imaging. This receptor is expressed on the surface of pro-inflammatory cells, including macrophages, dendritic cells, and activated T-cells. Yang et al. preclinical study demonstrated a high affinity of the RP to vulnerable atherosclerotic plaques and showed better results compared to ^18^F-FDG imaging of the same animals [87].

#### 2.2.12. Phagocytic Activity

Phagocytic activity of macrophages present in plaques can also be a target for molecular imaging to verify active atherosclerotic lesions.

For such procedures, nanoparticles have been developed, including those labeled with ^64^Cu-TNP (trimodal nanoparticles), which can also be detected using fluorescent and magnetic resonance technologies), which, according to Nahrendorf et al., concentrate in atherosclerotic plaques of experimental animals (mice) [88].

A similar tracer, consisting of ^18^F-labeled iron oxide nanoparticles and designed to detect macrophages, was previously developed by the same group of authors and used for vascular aneurysms [89]. However, information about its use in atherosclerotic lesions could not be found.

### 2.3. Plaque Formation

The fibrous tissue forms a capsule around the necrotic core of the plaque, and the part of this capsule located directly under the endothelium (at the border with the bloodstream) forms the plaque cap. The processes associated with the fibrous transformation of plaque are completed by the formation of a fibrous plaque (early fibroateroma)—the dominant substance of atherosclerosis. In addition to macrophage infiltration, atheroma also attracts other pro-inflammatory cells, in particular, T-lymphocytes [9,90].

#### Interleukin-2 Receptor

The interleukin-2 (IL-2) receptor overexpressed on activated T lymphocytes is a very attractive biomarker in assessing the vulnerability of plaques. Glaudemans et al. performed a visual analysis of scintigrams, and it revealed high uptake of a special RP affine to the IL-2 receptor, ^99m^Tc-HYNIC-IL-2, in seven out of ten symptomatic atherosclerotic plaques, as well as SPECT/CT, allowed visualization in eight out of ten cases [91]. A correlation was also found between the number of CD25+ lymphocytes and the total number of CD25+ cells in the plaque, on the one hand, and the ratio between the target accumulation in the plaques and background uptake in the adjacent carotid artery, on the other. Micro-SPECT showed selective uptake of ^99m^Tc-HYNIC-IL-2 in plaque components, excluding its lipid core [91].

Based on the presented data, it can be assumed that molecular imaging based on the binding of interleukin-2 receptors can be useful for verifying inflammatory processes in atherosclerotic plaques by the presence of activated T-lymphocytes in them. Annovazzi et al. came to a similar conclusion in their work with ^99m^Tc-labeled interleukin-2 [92].

### 2.4. Thin-Capsule Fibroatheromas

In people over the age of 50, thin-capsule fibroateromas can form in the walls of blood vessels. The development of inflammation in fibrous plaques is mediated by macrophages, which secrete a significant amount of pro-inflammatory cytokines, reactive oxygen species, and blood clotting factor III, which promotes migration of monocytes, T-lymphocytes, and neutrophils into the plaque. In addition, migration is also facilitated by small vessels sprouting into the plaque (from vasa vasorum). Activation of apoptotic processes in plaques leads to a violation of the structure of the plaque capsule and causes the risk of its destruction. In other words, the plaque becomes vulnerable.

#### 2.4.1. Neoangiogenesis

Neovascularization is considered one of the main factors of plaque vulnerability, and therefore specific imaging agents have been developed to detect newly formed vessels in the plaques. The target for such visualization is the integrin avβ3 expressed on macrophages, migrating smooth muscle cells, and endothelial cells in the vasa vasorum and intra-plaque microcirculation [93].

The tripeptide arginine-glycine-aspartic acid (RGD) has a high affinity for the integrin avβ3 and is often used as the main part of RPs targeting integrin. ^18^F-galacto-RGD has been shown to preferentially bind to damaged atherosclerotic plaques in the carotid arteries of patients within a few weeks after a stroke [94]. Saraste et al. performed experiments on mice, which showed that the use of ^18^F-galacto-RGD makes it possible to evaluate the effectiveness of treatment in atherosclerosis [95].

Among the disadvantages of ^18^F-galacto-RGD, the long time required for labeling is noted [93]. In this regard, the search and study of similar RPs, including ^18^F-Alphatide II and ^68^Ga-NOTA-PRGD2, have several advantages, including ease of preparation, fast labeling, and acceptable pharmacokinetics in vivo in comparison with most monomeric RGD peptides [96]. Another promising radiotracer is ^18^F-flotegatide, which also contains the RGD sequence [97].

Radiopharmaceuticals for the assessment of neoangiogenesis in plaques were also synthesized for SPECT. In particular, Vancraeynest et al. have shown that ^99m^Tc-maracyclatide allows in vivo identification of plaques with signs of inflammation in mice and, thus, provides the possibility of non-invasive detection of high-risk plaques [98]. Other dimeric RPs (^99m^Tc-IDA-D—[c(RGDfK)]_2_) also showed a high affinity for unstable atherosclerotic plaques, as it was reported by Sun Yoo et al. [99].

In addition to integrin, which is expressed not only on vascular cells, the vascular endothelial growth factor receptor, whose monoclonal antibodies were labeled with radioactive zirconium and tested in ex vivo experiments, can also be considered a target [100].

#### 2.4.2. Hypoxia

Hypoxia refers to signs of plaque vulnerability due to insufficient perfusion in the large necrotic nucleus. In preclinical and clinical studies, RPs targeted to hypoxia sites were studied, among which ^18^F-FMISO is the most well-known and commercially available FMISO. Selective plaque uptake of ^18^F-fluoromizonadazole was demonstrated in a rabbit model of atherosclerosis [101]. In clinical studies, uptake of the highly specific hypoxia marker ^18^F-HX4 by carotid artery plaques has been observed in individuals who have suffered TIA due to plaque rupture [102].

A clinical trial by Joshi et al. including the results of the use of ^18^F-FMISO PET in 16 patients with recent TIA or stroke showed that the uptake of the radiopharmaceutical was slightly higher in symptomatic plaques on the affected side than in the plaques in contralateral arteries (TBR of 1.11 ± 0.07 vs. 1.05 ± 0.06; *p* < 0.05) and demonstrated a correlation with the activity of ^18^F-FDG, which confirms the role of hypoxia in the launch and maintenance of inflammation in the plaque [103].

Another possible method for hypoxia imaging is the ^64^Cu-ATSM PET/MRI, which was studied for atherosclerosis-associated hypoxia. Its main purpose is to detect hypoxia in tumors because this neutral lipophilic RP crosses membranes easily and undergoes reduction only in hypoxic cells and becomes trapped in these cells, while it is washed out from the normoxic cells [104]. This RP’s applicability to the PET/MR imaging of hypoxic atherosclerotic sites was demonstrated by Nie et al. [105]. It was demonstrated that in a rabbit model, this RP is a promising agent that colocalizes with immunohistochemical markers of the hypoxia. Recently, another preliminary clinical study was published by Nie et al. [106]. It demonstrates that the tracer is applicable for atherosclerotic plaque imaging, and RP’s uptake corresponds to hypoxic macrophages within the lipid-rich core of the plaque. ^64^Cu has a longer half-life time (12.7 h) that makes longer imaging intervals possible, but on the other hand, the absorbed dose is also higher, which leads us to the need for rigorous analysis of its benefits and risks, which is yet to be done.

#### 2.4.3. Macrophage Death

^99m^Tc-labeled annexin V has a high affinity for phosphatidylserine, which is located on the membrane of apoptotic cells. This RP is mainly used in oncology, but reports are available on its use in heart failure, after heart transplantation, and in atherosclerosis [107]. In a study by Kietselaer et al., in four individuals undergoing carotid endarterectomy, RP uptake correlated with high-risk plaque characteristics (macrophage infiltration and hemorrhage in the plaque matrix), which justifies the potential use of labeled annexin V in the identification of unstable plaques [108].

Similar RPs that are tropic to the necrotic component in the plaque structure were also developed for PET. In particular, ^68^Ga-labeled annexin V is detected by molecular imaging in plaques with necrotic lesions, as well as in matrix vesicles containing hydroxyapatite in atherosclerosis, which is explained by the fact that cell death can be one of the important stimuli for microcalcification [109]. ^18^F-ML-10 (2-(5-fluoropentyl)—2-methylmalonic acid) is a radiotracer for positron emission tomography (PET), which accumulates in cells with apoptosis-specific membrane changes. Hyafil et al. reported a strong correlation between the level of accumulation of ^18^F-ML-10 in different aortic segments recorded by autoradiography and the number of apoptotic cells on histological sections corresponding to the above-mentioned aortic segments in a rabbit model of atherosclerosis [110]. In addition, Pang et al. published data suggest that ^18^F-ML-10 may be useful for quantifying the vulnerability of atherosclerotic plaques rich in apoptotic cells [96,111].

#### 2.4.4. Proteases

Vulnerable plaques are morphologically characterized by a thin fibrous cap covering the large lipid nucleus. Matrix metalloproteinases (MMPs) destroy the extracellular matrix that makes up the cap, which causes plaque destabilization and can be accompanied by the rupture of its cap. In addition, the above-mentioned LOX-1 also induces the expression and activation of MMP [112].

To visualize matrix metalloproteinases, RPs based on MMP inhibitors with radioactive labels were developed: ^99m^Tc-RP805 (MPI), ^111^In-RP782, etc., which, as was shown in experiments, demonstrate higher accumulation in atherosclerotic altered vessels of mice with atherosclerosis compared to mice with intact vessels [113]. In model experiments by Fujimoto et al., it was shown that ^99m^Tc-RP805 can also be used for dynamic monitoring of the state of a plaque during statin therapy [114]. Matrix metalloproteinase inhibitors were also labeled with other radionuclides, including positron-emitting ones, but these drugs were sparsely used [115].

In addition to specific MMP inhibitors, labeled antibodies to matrix metalloproteinases can also be used to visualize these molecules. In particular, a higher accumulation of ^99m^Tc-labeled monoclonal antibodies to MT1-MMP was demonstrated in large atheromas (grade IV) compared to neointimal changes or other more stable atherosclerotic lesions [116]. The authors of this study indicate that further research is required to implement RP in practice.

Another strategy for molecular imaging of MMP is the use of labeled proteinase substrates, but studies of these experimental preparations have been only partially successful due to the non-specific uptake of RP [112,117].

### 2.5. Unstable Plaques

#### 2.5.1. Calcification

Valuable data that is acquired during the molecular imaging procedures of the calcification process in clinical practice was discussed earlier [7].

#### 2.5.2. Thrombosis

Blood clot formation caused by plaque rupture is the most important mechanism leading to acute MI and sudden cardiac death, as well as ischemic strokes. Thrombotic atheromas are an extremely important prognostic target for ranking the risk of vascular events and ensuring timely and effective treatment. Blood clotting factor III (tissue factor or tissue thromboplastin) initiates an exogenous blood clotting cascade that leads to the formation of a blood clot in vivo. In atherosclerotic lesions, it was identified in several cell types, including endothelial, smooth myocytes, monocytes, macrophages, and foam cells, but its expression increased at later stages of atheroma development [109].

^99m^Tc-labeled monoclonal antibodies that have been proposed by Temma et al. can be used to visualize this type of molecule, and they showed six times more intense binding in the wall of the aorta affected by atherosclerosis in experimental animals compared to the control [118].

Since fibrin is localized in blood clots, rather than in circulating blood, the approach based on its visualization allows achieving high specificity in the detection of blood clots in particular, the EP-2104R molecule that consists of a short peptide that binds to fibrin and is conjugated with gadolinium as a contrast agent for MRI [119]. There is also information about a developed tracer with the radioactive label ^64^Cu-EP-2104R, which allows multimodal visualization of fibrin deposits in blood clots in rats [120].

In addition to the above data, there are isolated reports of the use of ^111^In-labeled platelets for the visualization of blood clots in atherosclerosis [121].

## 3. Discussion

In the modern scientific literature, there is a criticism of the theory of unstable plaques, which indicates that the finding of one or more vulnerable atheromas may indicate a higher stage of development of atherosclerotic lesions in general [122]. At the same time, it should be underlined that the presence of a vulnerable plaque is not sufficient for a vascular catastrophe. It must also be accompanied by a blood thrombosis tendency, without which the plaque will be able to recover from the damage without the formation of thrombotic mass. A higher metabolically active plaque load revealed with molecular imaging corresponds to a higher risk of capsule tear events and circulation decline in the presence of predisposition to thrombosis but not necessarily in the vascular territory of the artery, where the specific plaque is located [123].

^18^F-FDG has not been replaced by other RP in clinical practice to date as it has shown itself very efficient in detecting inflammatory processes in plaque structure, and therefore, its vulnerability. ^18^F-FDG has a benefit over other RPs in that it accumulates in a range of cells that express GLUT-type transporters, allowing for a high signal in the zone of active inflammation due to ^18^F-FDG pickup by macrophages, leukocytes, and activated smooth muscle cells. Some other RPs, being highly specific, are concentrated in a small area of the vascular wall near the lipid core of the plaque, as well as in the fibrous capsule and the adjacent adventitia. This area is, in general, too small for adequate visualization—in fact, it is much smaller than such inflammatory areas in more easily diagnosed vasculitis, when inflammatory cells infiltrate the entire thickness of the vessel wall for a significant length. Other tracers are much more selective and target links in the pathogenesis of atherosclerotic lesions, in particular, activated macrophages and their receptors, which causes a low accumulation of RP, which reduces the effectiveness of detecting atherosclerotic lesions in clinical practice. Many RPs developed in recent years need more significant evidence that their accumulation in atherosclerotic plaque indicates the progress of atherosclerosis and is associated with the risk of future cardiovascular disasters.

Another problem that is indirectly related to the high specificity and limited size and volume of the RP localization area is called partial volume error, which is associated with measurements of RP activity in the lesion, background activity in adjacent parts of the vessel wall, and (or) in adjacent tissues. All other things being equal, the formed area of interest for quantitative calculation will determine the accuracy of measurement since the latter depends not only on the intensity of RP accumulation but also on the size of the lesion. Therefore, if the affected area is small, when constructing the area of interest around it, the accuracy of calculating quantitative indicators may be extremely low due to the background accumulation of RP in intact adjacent tissues falling into the area of interest. This effect is especially pronounced during clinical imaging procedures, where the resolution of the scanner is relatively low and only large foci can be detected that exceed the resolution of the scanner by about three times (that is, in general, at least 10 mm) [124].

However, the use of specific RPs is still possible and should be aimed at obtaining information about the pathogenesis of the process and its links in vivo, the specifics of the response to treatment of atherosclerotic lesions, as well as in the development of new drugs. The main specific pathophysiologic mechanisms and RP are summarized in Table 1.

The issue of quantifying accumulation is still a subject of debate. Direct PET scanner measurements are expressed as RP activity in becquerels per milliliter of tissue volume. In oncological practice, the most used measure is a standardized uptake value (*SUV*), which is defined as the ratio of RP accumulation in the analyzed lesion to the expected accumulation with a uniform distribution of the administered dose in the human body volume. The equation for *SUV* normalized to body weight (*SUVbw*) is as follows:

(1) $$SUVbw\left(t\right)=\frac{C_{img}\left(t\right)}{\left(ID/BW\right)}$$

where *C_img_* stands for the radioactivity measured from an image acquired at a time (*t*) decay corrected to *t* = 0, expressed as volume concentration (Bq/mL),

*ID* is an injected dose (in Bq), and *BW* is body weight in kg.

Lean body mass may be used instead of body weight as well.

However, applying this indicator directly in the vessel wall assessment revealed that meaningful distinction of plaques with and without evidence of inflammation was impossible, necessitating the use of another alternative estimated value: Target-to-background ratios (TBR) [125]. An unmodified ipsi- or contralateral arterial wall or blood pool can be used as the background in this method.

Due to a decrease in the activity of RP in blood plasma over time, and therefore, an increase in the target-to-background ratio, estimating the ratio of accumulation in the focus to the radioactivity in the blood pool leads to mistakes. When SUV is used as a measurement tool, this error does not occur. The clearance of the injected RP from the blood is slower in patients with poor renal function, resulting in a drop in the computed target-to-background activity ratio, which is still the most widely used evaluation approach in the field of vascular molecular imaging [125].

The research results published so far are characterized by a pronounced heterogeneity of measurement methods (essentially different SUV and TBR), a small number of groups, insufficiently strict selection criteria, or their relativity (in works studying vascular problems simultaneously with tests for verification and staging of oncological diseases), special preparation of the included individuals do not allow for a full analysis and comparison of the results, which, in turn, significantly limits the information regarding reproducibility. Therefore, it is expected that efforts will be undertaken in the future to unify the relevant protocols in order to assure comparability of the results of RP tests aiming to assess atherosclerotic lesions. Otherwise, generalizing conclusions will be equally ambiguous, and the feasibility of employing the suggested approaches in practice would be questioned.

To date, there are a limited number of studies that directly compare atherosclerosis-specific tracers with each other. This makes it difficult to assess the advantages and disadvantages of each. Published studies focus primarily on comparing the accumulation of a radiopharmaceutical with the intensity of the pathophysiological process occurring in the plaque structure. A related limitation is the resolution of scanners used in the discussed studies of specific RPs. Generally, those are the specialized devices for preclinical research on animals with relatively high spatial resolution. However, scanners used in clinical practice are of lower resolution, and many of the changes described may be too small to be visualized with it. It leads to the predominantly research nature of developments aimed at shedding light on the pathogenesis of the disease and not introducing them into clinical practice.

The economic and regulatory complexity of registering new drugs for human use is another factor in the low amount of highly specific RPs on the market. Talking about economic feasibility, for manufacturing companies, the development of tracers specific only to atherosclerotic lesions is risky. From this point of view, it is much more effective to search for and create RPs that are targeted to cells (or processes) involved in the implementation of pro-inflammatory reactions not only in vascular pathology (in particular, in atherosclerosis) but also in other conditions and diseases, primarily cancer. For example, RPs such as ^111^In-oxine and ^99m^Tc-tilmanocept for SPECT have a wide range of applications other than atherosclerotic plaque imaging. In the PET studies, TSPO-targeted RPs (^11^C-PK11195 and ^18^F-GE-180) are the most promising agents because they can also be applied for inflammation imaging in the brain and other sites.

The high cost of producing highly specific RP for vascular lesions does not allow the widespread implementation of the methods for molecular visualization of their distribution in clinical practice. In the case of using them for scientific purposes, it requires the researcher to be very careful in interpreting the results of determining the value of additional clinical information obtained.

## 4. Conclusions

Atherosclerotic plaque instability is considered to be a crucial component in the pathogenesis of the primary and most significant events that emerge in this case—acute coronary syndrome and myocardial infarction, as well as ischemic infarction.

Various differences in definitions, nomenclature, and descriptions of the observed phenomena suggest that scientists have yet to reach a consensus, and the clear “danger” of atherosclerotic plaque remains a critical problem in many adjacent fields of clinical and biological expertise. As a result, inconsistency in approaches to the detection of high-risk plaques emerges.

Molecular imaging, unlike other radiological methods, is most susceptible to existing uncertainties since it allows you to detect processes, their consequences, or results that are part of or associated with atherosclerosis.

An analysis of the available literature suggests that one of the main processes that accompany or are part of atherosclerosis and, apparently, mediate its activity and vulnerability to atheroma, in which, in the opinion of researchers, it is reasonable to use molecular imaging, is inflammation.

Highly specific RPs that are targeted to macrophage receptors, or other molecules expressed on the cell surface, and enzymes (primarily metalloproteinases) allow us to clarify and expand the understanding of various links in the pathogenesis of vascular lesions and assess the effectiveness of treatment with highly specific, targeted drugs. Currently, such studies, which undoubtedly have a high scientific value, cannot be implemented in clinical practice, which is due, on the one hand, to the high cost of producing RP, and on the other, to the limitations of the evaluation tools used. Among many radiopharmaceuticals reviewed in this article, the most promising ones are ^111^In-oxine and ^99m^Tc-tilmanocept for SPECT and TSPO-targeted RPs for PET.

The presented data indicate an increasing interest in the results of molecular imaging with highly specific RPs, most likely associated with obtaining new information about the patterns and ways of implementing atherosclerosis as the main involutional process accompanying modern humans. Thus, nuclear medicine research, which is regarded today as hardly applicable in practice, can be reasonably recognized as one of the most advanced in modern biomedicine.

## Figures and Tables

**Table 1:** The main pathophysiological mechanism used for atherosclerotic plaque imaging at each stage.

Stage of a Plaque	Target Pathophysiological Process	RP
Thickening of intima, intimal xanthoma, or “fatty streak”	Lipoprotein accumulation	^99m^Tc, ^111^In, ^68^Ga-labeled LDL ^89^Zr-labeled HDL
	Lipoprotein oxidation	^131^I and ^125^I-labeled antibodies to OSE ^89^Zr-LA25
	Monocyte adhesion and infiltration	VCAM-1: ^18^F-4V; ^99m^Tc- or ^18^F labeled cAbVCAM1-5 P-selectin: ^64^Cu-antibodies, ^68^Ga-fucoidan
Macrophage migration and “foam cell” formation, macrophage activation	Native cells migration	^111^In-oxine, ^99m^Tc-exmetazime blood cell labelling
	Increased macrophage glucose uptake and metabolism	^18^F-FDG *
	Increased membrane production	^18^F-choline *
	Scavenger receptor	^99m^Tc-specific mab to LOX-1
	Somatostatin receptor	^68^Ga-DOTA-TATE * ^111^In-DOTA-JR11
	Mannose receptors	^68^Ga-mannose-specific mab ^99m^Tc-tilmanocept * ^18^F-FDM ^68^Ga-NOTA-MSA
	Folate receptor	^99m^Tc-EC20 (etarfolatide) ^18^F-fluorofolic acid
	TSPO	^11^C-PK11195 ^18^F-GE-180 ^11^C-PBR28
	Chemokine receptor	^64^Cu-DOTA-vMIP-II ^68^Ga-pentixaphor
	LFA-1	^111^In-DOTA-butylamino-NorBIRT (DANBIRT)
	Nicotinic acetylcholine receptor	^18^F-ASEM
	Increased phagocytosis	^64^Cu-trimodal nanoparticles ^18^F-iron oxide nanoparticles
Plaque formation	Interleukin-2 receptor	^99m^Tc-HYNIC-IL-2
	Neoangiogenesis	^18^F-galacto-RGD ^18^F-Alphatide II ^68^Ga-NOTA-PRGD2 ^18^F-flotegatide ^99m^Tc-maracyclatide ^99m^Tc-IDA-D—[c(RGDfK)]2
	Hypoxia	^18^F-FMISO * ^18^F-HX4
	Macrophage apoptosis	^99m^Tc- and ^68^Ga-labeled annexin V
	Proteases	^99m^Tc-RP805 (MPI), ^111^In-RP782
Plaque progression and deterioration	Calcification	^18^F-sodium fluoride *
	Thrombosis	^99m^Tc-labeled mab to tissue thromboplastin ^111^In-labeled platelets

RP—radiopharmaceutical, LDL—low-density lipoproteins, HDL—high-density lipoproteins, OSE—oxidation specific epitopes, VCAM—vascular cell adhesion molecule, mab—monoclonal antibody, LOX-1—lectin-like receptor for oxidized low-density lipoproteins, FDG—fluorodeoxyglucose, FDM—fluoro-D-mannose, NOTA-MSA—human serum albumin enriched with mannose, TSPO—18 kDa translocation protein, LFA-1—the lymphocyte-function associated antigen-1, RGD—arginine-glycine-aspartic acid. (*) RPs denoted with an asterisk are readily achievable for clinical practice.

## Data Availability

All data is available from corresponding author or A.K. on a reasonable request.
